# EARLY FUNCTIONAL FACTORS FOR PREDICTING OUTCOME OF INDEPENDENCE IN DAILY LIVING AFTER STROKE: A DECISION TREE ANALYSIS

**DOI:** 10.2340/jrm.v56.35095

**Published:** 2024-05-07

**Authors:** Heegoo KIM, Chanmi LEE, Nayeong KIM, Eunhye CHUNG, HyeongMin JEON, Seyoung SHIN, MinYoung KIM

**Affiliations:** 1 Department of Rehabilitation Medicine, CHA Bundang Medical Center, CHA University School of Medicine, Seongnam, Republic of Korea; 2 Digital Therapeutics Research Team, CHA Future Medicine Research Institute, Seongnam, Republic of Korea; 3 Rehabilitation and Regeneration Research Center, CHA University School of Medicine, Seongnam, Republic of Korea

**Keywords:** stroke, prediction, activities of daily living, motor function, cognition

## Abstract

**Objective:**

This study aimed to investigate the predictive functional factors influencing the acquisition of basic activities of daily living performance abilities during the early stages of stroke rehabilitation using classification and regression analysis trees.

**Methods:**

The clinical data of 289 stroke patients who underwent rehabilitation during hospitalization (164 males; mean age: 62.2 ± 13.9 years) were retrospectively collected and analysed. The follow-up period between admission and discharge was approximately 6 weeks. Medical records, including demographic characteristics and various functional assessments with item scores, were extracted. The modified Barthel Index on discharge served as the target outcome for analysis. A “good outcome” was defined as a modified Barthel Index score ≥ 75 on discharge, while a modified Barthel Index score < 75 was classified as a “poor outcome.”

**Results:**

Two classification and regression analysis tree models were developed. The first model, predicting activities of daily living outcomes based on early motor functions, achieved an accuracy of 92.4%. Among patients with a “good outcome”, 70.9% exhibited (*i*) ≥ 4 points in the “sitting-to-standing” category in the motor assessment scale and (*ii*) 32 points on the Berg Balance Scale score. The second model, predicting activities of daily living outcome based on early cognitive functions, achieved an accuracy of 82.7%. Within the “poor outcome” group, 52.2% had (*i*) ≤ 21 points in the “visuomotor organization” category of Lowenstein Occupational Therapy Cognitive Assessment, (*ii*) ≤ 1 point in the “time orientation” category of the Mini Mental State Examination.

**Conclusion:**

The ability to perform “sitting-to-standing” and visuomotor organization functions at the beginning of rehabilitation emerged as the most significant predictors for achieving successful basic activities of daily living on discharge after stroke.

LAY ABSTRACT

In stroke rehabilitation, the prediction of activities of daily living is crucial for establishing effective therapeutic goals. We analysed data from 289 patients undergoing rehabilitation, encompassing all measured functional assessments, including item scores. The target outcome was defined as the modified Barthel Index, an indicator of activities of daily living performance, at the point of discharge. Two models were developed. The first is based on early motor function, which revealed that higher scores in the “sitting-to-standing” category on the motor assessment scale, higher Berg Balance Scale scores, and younger age were predictive of more favourable activities of daily living outcomes (92.4% accuracy). The second model, based on early cognitive function, demonstrated that lower scores in the “visuomotor organization” category of the Lowenstein Occupational Cognitive Assessment and a lower “time orientation” scores in the Mini Mental State Examination were indicative of suboptimal activities of daily living outcomes (82.7% accuracy). In conclusion, the ability to perform sitting-to-standing and exhibiting sound visuomotor organization stands out as crucial in predicting post-stroke activities of daily living outcomes. Clinicians can integrate these early functional assessments into their decision-making processes when establishing therapeutic goals.

Stroke remains a leading cause of long-term disability, significantly impacting quality of life (QoL) for individuals and their families (1), extending its consequence to areas such as social participation (2) and the ability to return to work (3). Notably, dependency in activities of daily living (ADL) is a prevalent consequence of stroke, persisting in approximately 35% of the stroke population during the first year post-stroke (4).

Given that stroke has emerged as a leading cause of basic ADL dependence, there is a significant value in exploring factors not only associated with stroke occurrence but also those determinants contributing to dependency in ADL. Previous studies have illuminated various factors linked to increased dependency in ADL among stroke survivors (5, 6). These factors encompass a range of considerations, including older age (7), comorbidities (7), and the severity of neurological impairments at stroke onset (8). A crucial point in rehabilitation is a “sensitive period” characterized by enhanced neuroplasticity in patients, where the majority of recovery from impairments occurs within the initial 6 months after a stroke (9). During this golden period of rehabilitation therapy, the focus of treatment shifts towards specialized medical intervention and intensive rehabilitation to enhance both motor and cognitive functions, directly associated with the ability to perform ADL (10). During the period of intensive rehabilitation care after a stroke, the improvement in basic ADL stands as a primary goal of rehabilitation and other medical interventions (11).

Accurate prediction of functional outcomes during the early recovery phase is of great consequence in stroke rehabilitation, facilitating evidence-based clinical decision-making and the establishment of realistic therapeutic goals (12, 13). Recent studies on stroke patients have investigated various approaches in predicting independence in ADL after stroke, with a focus on early functional factors. Previous research has indicated that trunk control impairment, assessed through the trunk impairment score for patients with acute stroke, is a robust predictor of ADL on discharge (14). Additionally, in a previous prospective multicentre cohort study, physical performance and general cognitive function emerged as the most reliable predictors of changes in ADL after stroke (15). In the realm of clinical practice, particularly at the beginning of rehabilitation, determination of goals for stroke patients remains a crucial yet challenging endeavour. However, there are few literature and clinical guidelines regarding specific variables that can be referenced to make these decisions. Furthermore, while the initial neurological functions have been acknowledged as potentially significant, no universally accepted score values of the commonly utilized functional assessments were known to predict the outcome of ADL after stroke.

For the development of clinical prediction rules, intended to assist clinicians in decision-making, various statistical techniques such as logistic regression and neural networks have been utilized (16). Among these methods, the Classification and Regression Analysis Tree (CART) stands out as a distribution-free regression method that constructs a tree by recursively partitioning the data into increasingly homogeneous subgroups, aiming to maximize the explained variance within each subgroup. At each stage (node), the CART algorithm identifies the explanatory variable and splitting value that best discriminates between 2 outcome classes. A full CART algorithm continues to add nodes until they become homogeneous or contain few observations (17). Consequently, the utility of the CART algorithm in building new prognostic models is on the rise in biomedical and clinical research (18).

In the current study, we aimed to investigate the specific predictive factors by developing separate models using the CART algorithm, encompassing stroke-related risk factors as well as early motor and cognitive functions, to determine the outcome of basic ADL on discharge in stroke patients.

## METHODS

### Design

This retrospective study was conducted on patients admitted to the Rehabilitation Medicine Department of a university-affiliated medical centre (CHA Bundang Medical Center, CHA University, Bundang, Republic of Korea). The rehabilitation medicine department of the university-affiliated medical centre in the present study is a key component of the institution’s commitment to comprehensive patient care. It offers intensive rehabilitation services with multidisciplinary teams and specialized programmes for stroke patients. Furthermore, approaches to novel clinical trials and research studies have been done in collaboration between the medical centre and university. The study protocol received approval from the Investigational Review Board of the Hospital Ethics Committee (2021-11-020) and was duly registered at ClinicalTrials.gov (NCT05898815).

### Study population and data collection

**Fig. 1:**
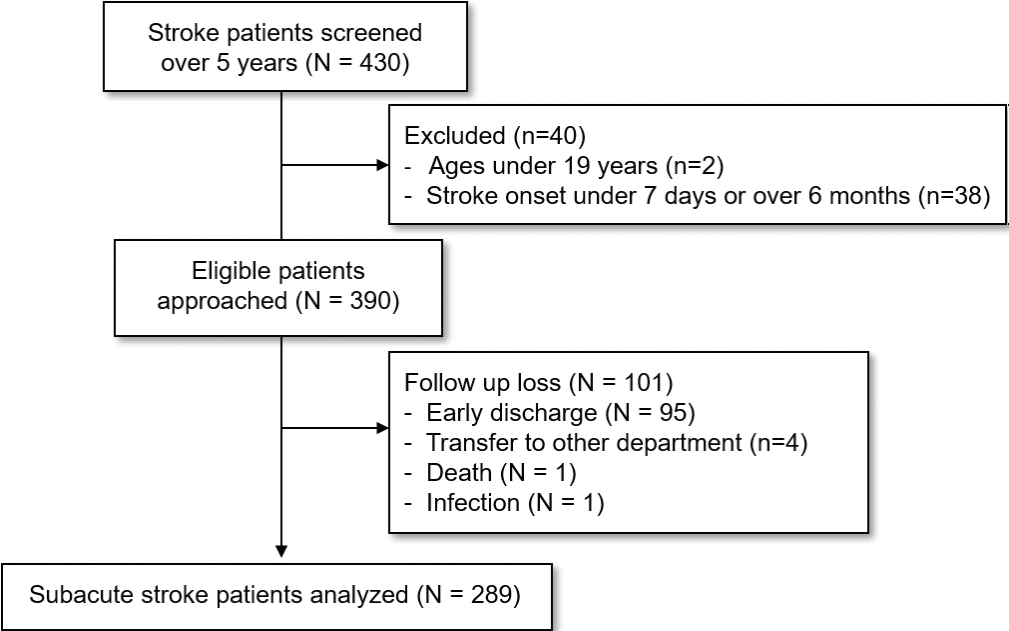
Trial profile.

Data were collected retrospectively on patients with stroke who underwent intensive rehabilitation after management of stroke care in the acute phase. The study population consisted of patients who were hospitalized for intensive rehabilitation following a completed stroke in the Rehabilitation Unit of CHA Bundang Medical Center between January 2017 and December 2022. These individuals underwent a comprehensive rehabilitation programme during a standard 6-week hospitalization period and underwent functional assessments on both admission and discharge, as per the routine clinical practice directed by physicians. The initial screening of patients was conducted by a dedicated team consisting of rehabilitation physicians who were responsible for reviewing medical records to identify potential study participants. The primary purpose of this initial screening was to create a preliminary pool of patients who met the basic criteria of having undergone intensive rehabilitation after stroke. Inclusion criteria encompassed individuals diagnosed with either ischaemic or haemorrhagic stroke based on brain computed tomography and magnetic resonance imaging findings, and who received comprehensive rehabilitation treatment. Exclusion criteria comprised individuals aged ≤ 19 years, and those whose stroke onset duration was either ≤ 7 days or > 6 months. According to the inclusion and exclusion criteria of our study, our research team selected data by analysing previously collected data in the rehabilitation unit between January 2017 and December 2022 following initial screening. Trial profile was described in Fig. 1.

### Clinical assessments

Clinical assessments on admission and discharge were conducted by a team of certified rehabilitation therapists. These therapists, who are part of the multidisciplinary team in the rehabilitation medicine department, have undergone specialized training to ensure high inter-rater reliability in administering and scoring the assessment tools used in this study. Before conducting functional measurements, assessors established inter-rater reliabilities for the tests, ensuring an intra-class correlation coefficient of > 0.9. Reliability for the measurements was routinely confirmed annually and for every new assessor enrolment. In instances of under-qualification, assessors were required to undergo institutional training programs and repeat the reliability test until meeting the stipulated requirement. Details of the clinical assessments are described in Appendix S1.

### Independent variables

The potential predictors considered in this study encompassed patient demographics, stroke characteristics, and motor and cognitive functioning on admission. Patient demographics and stroke characteristics included sex, age, type of stroke (haemorrhagic or ischaemic), side of hemiparesis, stroke-related risk factors such as hypertension, diabetes mellitus, atrial fibrillation, hyperlipidaemia, history of stroke, and the time since stroke onset. Motor predictors included range of motion (ROM), muscle strength of each joint graded with manual muscle test (MMT), motor assessment scale (MAS), Berg Balance Scale (BBS), Rivermead Mobility Index (RMI), trunk impairment scale (TIS), functional ambulatory category (FAC), and Fugl-Meyer Assessment of Upper Extremity (FMA-UE). For motor assessments, potential predictors included each measured joint angle for ROM, MMT, subscales of 8 items of MAS, subscales of the 3 categories of TIS, and subscales of the 4 sub-items of FMA-UE. Total scores for BBS and RMI were included as independent variables. In cognitive assessments, potential predictors comprised each score in sub-items of the Mini Mental State Examination (MMSE) and Lowenstein Occupational Therapy Cognition Assessment (LOTCA). Details are described in Appendix S1.

### Dependent variable

The final outcome was assessed using the modified Barthel index (MBI), a functional assessment tool comprising 10 items designed to measure basic ADL outcomes. The items encompass feeding, personal hygiene (grooming), bathing, dressing, toilet transfer, bladder control, bowel control, chair/bed transfers, stair climbing, and ambulation (19). The MBI employs 3 different rating scales: 0–5 for bathing and personal hygiene; 0–10 for feeding, dressing, toilet transfer, bladder control, bowel control, and stair climbing; and 0–15 for chair/bed transfers and ambulation. The total MBI score ranges from 0 to 100, with higher scores indicative of a greater degree of independence in performing ADLs. Patients were classified into 2 groups based on their MBI scores on discharge: those with a score ≥75 were classified into the “good outcome” group, while those with a score < 75 were categorized into the “poor outcome” group (20).

### Statistical analysis

Data analyses were conducted using IBM SPSS Statistics software version 25 (IBM Corp, Armonk, NY, USA). The normality of the distribution of basic characteristics was assessed using the Shapiro–Wilk test. The statistical differences in basic characteristics and the severity of functions on admission between the 2 groups were determined using the χ^2^ test for categorical variables and the Mann–Whitney *U* test for continuous variables. Statistical significance was set at *p* < 0.05.

Subsequently, a classification model was developed using CART analysis, grounded on a binary recursive stratification strategy (21). The dependent variable was partitioned into 2 homogeneous subgroups (binary). The forward approach was employed for CART to identify the independent variable and its associated cut-off value, resulting in the most effective split. The Gini Index was improved to measure homogeneity within and heterogeneity between subgroups, guiding the determination of the most effective split (22). The process of splitting continued until patients within subgroups had a similar endpoint (Gini Index ≤ 0.01) or the subgroups became too small for further splitting (21). Samples with missing values for the most effective splitting variable were classified using the most appropriate surrogate variables.

To assess our model’s performance in classifying stroke patients into “good outcome” and “poor outcome” groups for basic ADL recovery, we employed multiple metrics: specificity, sensitivity, positive predictive value, negative predictive value, and overall accuracy. These metrics enabled a well-rounded evaluation, highlighting not only the model’s accuracy but also its clinical relevance by effectively identifying patients’ likely ADL outcomes. To compute adjusted overall accuracy, a 5-fold cross-validation process was employed. The sample was randomly divided into 5 subsets, with one subset designated as the validation set and the remaining subsets utilized for training in each iteration of the 5-fold cross-validation. This process was repeated for each subset, and the average misclassification error was calculated to evaluate model performance. We developed 3 distinct CART models to investigate the predictive functional factors influencing the acquisition of basic ADL recovery during the early stages of stroke rehabilitation. The first and second models were constructed to predict ADL outcomes based solely on motor or cognitive function evaluation results, respectively, on rehabilitation admission. The decision to construct those 2 separate models stemmed from our objective to explore motor and cognitive functional predictors independently. The third model integrated both motor and cognitive function assessments on admission to assess combined effect both of motor and cognitive function on rehabilitation admission.

## RESULTS

### Baseline descriptions

A total of 430 stroke patients who were hospitalized between 2017 and 2022 were screened, and 289 individuals were identified to meet the inclusion criteria. Following an assessment on discharge, 86 patients (29.8%) were categorized as having achieved a “good outcome” (MBI score ≥ 75), while 203 patients (70.2%) were categorized with a “poor outcome” (MBI score < 75). The baseline assessments of stroke severity in the total number of patients revealed that the majority exhibited moderate to severe impairment according to the results in upper extremity score of FMA (23, 24), MMSE (25), and FAC (26). Comparative analyses revealed significant distinctions within the good outcome group, including younger age, shorter interval from stroke onset to the initiation of rehabilitation, and reduced length of stay in the rehabilitation unit (χ^2^ test or paired *t*-test, *p* < 0.05). Additionally, the scores of MAS, FMA-UE, MMSE, and K-MBI on admission were higher in the good outcome group (Mann–Whitney *U* test, *p* < 0.05). Stroke-related risk factors including hypertension, diabetes, atrial fibrillation and hyperlipidaemia were not chosen by the CART algorithm of our 3 models due to their comparative lack of predictive power. The baseline patient characteristics are outlined in Table I.

**Table I:** Demographic information of participants

	Total	Groups by outcome of K-MBI at discharge		*p*-value
		Good outcome	Poor outcome	
Demographic characteristics, *n* (%)	289 (100)	86 (29.8)	203 (70.2)	
Age in years, median (IQR)	63.0 (53.0–72.0)	54.5(49.0–64.0)	65.0 (56.0–75.0)	< 0.001**
Male, *n* (%)	164 (56.7)	55 (64.0)	109 (53.7)	0.108
Onset duration in days, median (IQR)	27.0 (16.0–79.6)	21.0 (13.0–46.2)	30.0 (19.5–88.0)	< 0.001**
Stroke type (infarctio:haemorrhage)	143:146	54:32	89:114	0.003**
Hemi side (R:L:both)	106:110:73	36:29:21	70:81:52	0.464
Recurrence rate, *n* (%)	51 (27.0)	16 (18.6)	35 (17.2)	0.781
Length of stay in rehabilitation unit by days, median (IQR)	49.0 (42.0–71.0)	43.0 (39.0–50.8)	51.0 (43.0–62.0)	0.003**
Risk factors, *n* (%)				
Hypertension	184.0 (63.7)	51.0 (59.3)	133 (65.5)	0.315
Diabetes	69.0 (23.9)	17 (19.8)	52 (25.6)	0.286
Atrial fibrillation	31.0 (10.7)	7 (8.1)	24 (11.8)	0.355
Hyperlipidaemia	32.0 (11.1)	12 (14.0)	30 (36.5)	0.310
Baseline stroke severity, median (IQR)				
Motor assessment scale	19 (5–46)	43 (34–46)	10 (3–21)	< 0.001**
Upper extremity Fugl-Meyer scale score	23 (4–57)	62 (44–66)	7 (4–39)	< 0.001**
MMSE score	16 (6–25)	25 (16–30)	12 (2–21)	< 0.001**
FAC score	1 (0–2)	3 (2–4)	1 (0–1)	< 0.001**
MBI score	32 (10–54)	63 (47–77)	18 (6–36)	< 0.001**

R: right; L: left; MMSE: Mini Mental State Examination; MBI: modified Barthel Index.

** Significant difference between good and poor outcome of MBI at discharge (Mann-Whitney *U* test or χ^2^ test, *p < 0.05, **p < 0.01).

### Results of Classification and Regression Analysis Tree for the first model, predicting ADL on discharge based on motor function on rehabilitation admission

**Fig. 2:**
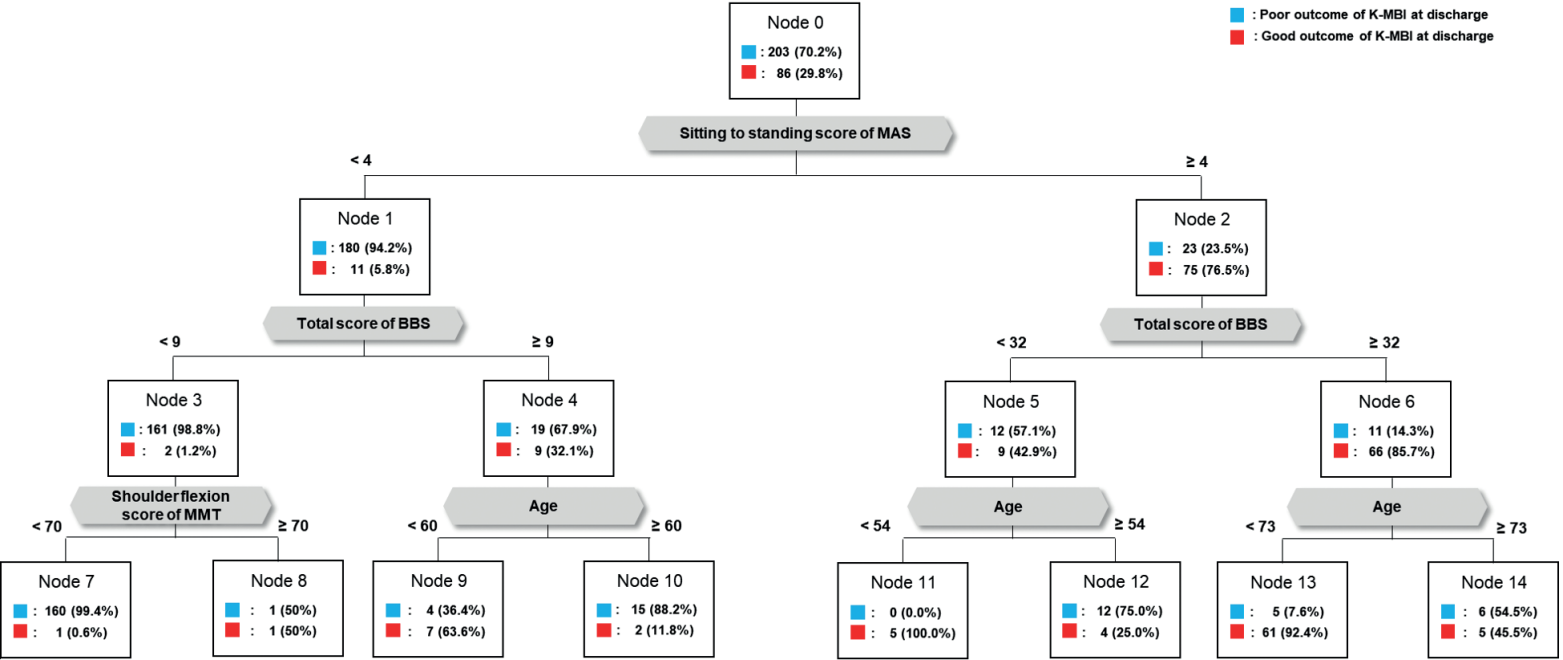
Model I for predicting outcome of ADLs on discharge by early motor functions. MAS: motor assessment scale; BBS: Berg balance scale; MMT: manual muscle test.

The first CART model for predicting ADL on discharge based on motor function metrics at the onset of rehabilitation (Model I) is illustrated in Fig. 2. The “sitting-to-standing” score in MAS emerged as the most influential predictor for ADL outcomes. Subsequently, the CART algorithm classified subgroups based on the sitting-to-standing score in MAS, distinguishing between scores < 4 points and scores ≥ 4 points. For patients with sitting-to-standing scores in MAS < 4 points, further classification was determined using a 9-point score in BBS. Within this subgroup, patients with sitting-to-standing scores in MAS < 4 points and BBS scores < 9 points were further divided based on the “shoulder flexor” score in MMT with a threshold of 70 points. Patients with sitting-to-standing scores in MAS < 4 points and BBS scores ≥ 9 points were classified into 2 subgroups based on an age threshold of 60 years.

Patients with a sitting-to-standing score in MAS ≥ 4 points were further categorized based on a BBS score of 32 points. Subsequently, patients with a sitting-to-standing score in MAS ≥ 4 points and a BBS score < 32 points were further classified based on an age criterion of 54 years. Patients with sitting- to-standing score in MAS ≥ 4 points and a total BBS score ≥ 32 were subdivided based on an age threshold of 73 years. Within the subgroup demonstrating a “good outcome” group in Model I, 70.9% exhibited (*i*) a sitting-to-standing score in MAS of ≥ 4 and (*ii*) a BBS score ≥ 32 points. The overall accuracy of Model I was 92.4%, and an overview of its performance is presented in Table II.

**Table II:** Model performances of the classification trees

Model performance	Model I	Model II
Sensitivity	0.85	0.63
Specificity	0.96	0.91
Positive predictive value	0.89	0.75
Negative predictive value	0.94	0.85
Overall accuracy	0.92	0.83

### Results of Classification and Regression Analysis Tree for the second model, predicting ADL on discharge based on cognitive function on rehabilitation admission

**Fig. 3:**
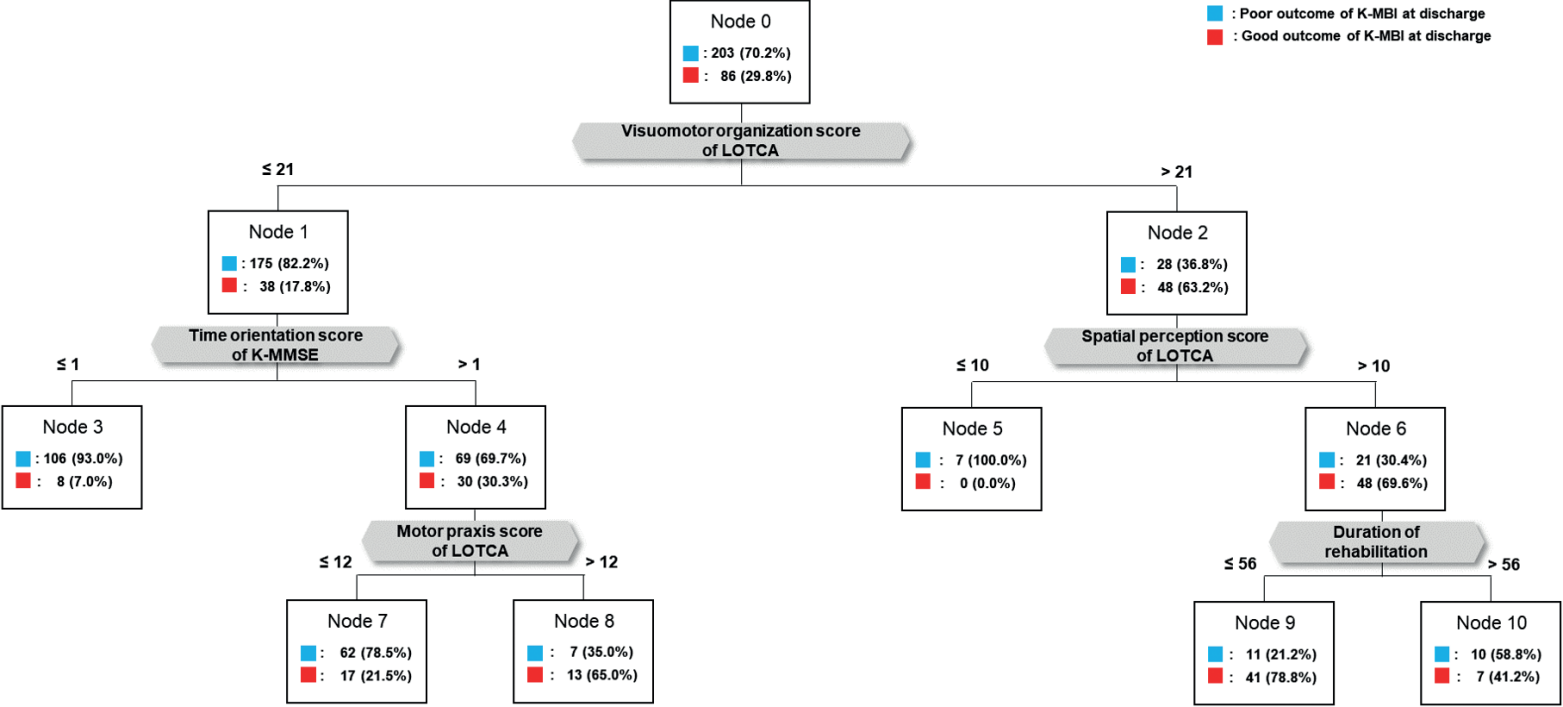
Model II for predicting outcome of ADLs on discharge by early cognitive functions. LOTCA: Lowenstein Occupational Therapy Cognitive Assessment; MMSE: Mini Mental State Examination.

The second CART model (Model II) for predicting ADL on discharge focused on cognitive function on rehabilitation admission, and is depicted in Fig. 3. The primary decision point in this model was identified as the “visuomotor organization” score in LOTCA. An optimal threshold of 21 points in visuomotor organization in LOTCA was established as the standard for determining the ADL outcome. Subsequently, patients with a visuomotor organization score in LOTCA ≤ 21 points were classified into 2 subgroups based on the “time orientation” score in the MMSE. Among these patients, those with a visuomotor organization score ≤ 21 points and a time orientation score in MMSE > 1 point were further classified based on the “motor praxis” score in LOTCA, with a threshold of 12 points.

Patients with a visuomotor organization score in LOTCA > 21 points were further categorized based on the “spatial perception” score in LOTCA, with a threshold of 10 points. Subsequently, patients with a visuomotor organization score in LOTCA > 21 points and a spatial perception score > 10 were further classified based on the “length of stay in the rehabilitation unit”, with a threshold of 56 days. Notably, within the “poor outcome” group in Model II, 52.2% exhibited (*i*) ≤ 21 points in the visuomotor organization category of LOTCA and (*ii*) ≤ 1 point in the time orientation’ category of the MMSE. The overall accuracy of Model II in selecting cognitive predictors for outcome of ADL was 82.7%. The detailed performance metrics of Model I is presented in Table II.

### Results of Classification and Regression Analysis Tree for the third model, predicting ADL on discharge based on both motor and cognitive function on rehabilitation admission

The third CART model for predicting ADL on discharge based on both motor and cognitive function metrics at the onset of rehabilitation (Model III) is illustrated in Fig. S1. The CART algorithm classified subgroups based on the sitting-to-standing score in MAS, distinguishing between scores < 4 points and scores ≥ 4 points. For patients with sitting-to-standing scores in MAS < 4 points, further classification was determined using a 9-point score in BBS. Within this subgroup, patients with sitting-to-standing scores in MAS < 4 points and BBS scores < 9 points were further divided based on the shoulder flexor score in MMT with a threshold of 70 points. Patients with sitting-to-standing scores in MAS < 4 points and BBS scores ≥ 9 points were classified into 2 subgroups based on an age threshold of 56 years.

Patients with a sitting-to-standing score in MAS ≥ 4 points were further categorized based on “orientation” score in LOTCA of 15 points. Subsequently, patients with a sitting-to-standing score in MAS ≥ 4 points and a orientation score in LOTCA < 15 points were further classified based on “upper extremity function” score in MAS of 6 points. Patients with sitting- to-standing score in MAS ≥ 4 points and a orientation score of LOTCA ≥ 32 were subdivided based on an attention score of 3 points. The overall accuracy of Model I was 93.1%, and an overview of its performance is presented in Table SI.

## DISCUSSION

In this study, the CART algorithm was employed to investigate early motor and cognitive functional factors at the onset of rehabilitation, predicting basic ADL outcomes on discharge. Our findings highlight the pivotal role of the ability to perform sitting-to-standing and visuomotor organization functions at the commencement of rehabilitation as the most significant indicators for determining basic ADL outcomes.

In Model I of this study, 70.9% of patients categorized as having a good outcome exhibited (*i*) a sitting-to-standing score in the MAS of ≥ 4 points out of 6 points of full scores in the sitting-to-standing category and (*ii*) a BBS score ≥ 32 points out of 56 points of full score. The importance of the sitting-to-standing movement is recognized as a crucial prerequisite for independent functioning, including standing from a chair, toilet, or bed, and walking (27). Previous studies have suggested the significance of sitting-to-standing as one of the most physically demanding movements, associated with various impairments such as muscle weakness, impaired balance (28), trunk control (29), and emotional factors such as anxiety (30). Our findings align with earlier studies indicating that the abilities to remain sitting and to stand up at 10 days after stroke onset strongly predict ADL function on discharge (31). Furthermore, a longitudinal cohort study demonstrated that the achievement of the ability to perform sitting-to-standing mostly occurred during the first 12 weeks after stroke onset, followed by improvements in gait speed and ADL (27). A novel aspect of our finding is the proposed cut-off value of 3 points in the sitting-to-standing score in MAS at the beginning of the rehabilitation as the first decision point determining basic ADL outcomes on discharge. This result underscores the importance of inpatient physical therapy focused on the specific ability to achieve independence in basic ADL. Additionally, the BBS score emerged as a predictor for achieving independent gait, strongly linked to ADL independence after stroke in previous studies (32). According to one study, for non-ambulatory early stroke patients, a BBS score of 13 points on admission was suggested as a cut-off predicting independent gait ability at 3 months post-stroke (32). In Model I of this study, patients with sitting-to-standing scores in MAS ≥ 4 points were further classified based on a BBS score of 32 points, exceeding the cut-off scores determining gait independence in previous reports. This may be attributed to the fact that these patients already scored ≥ 4 points in sitting-to-standing in MAS, indicating relatively proficient balance functions. Moreover, the higher cut-off score in BBS in our study, compared with previous studies with findings focused on predicting ambulation ability, emphasizes the demand for higher balancing capacity to guarantee independent ADL. In summary, Model I of our study highlights that, at the commencement of rehabilitation, the ability to perform sitting-to-standing movements and balance function are critical prerequisites for achieving independence in ADL after a stroke.

In Model II of our study, within the poor outcome group, 52.2% exhibited (*i*) ≤ 21 points in the visuomotor organization category in LOTCA out of 28 points of full subscore and (*ii*) ≤ 1 point in the time orientation category in MMSE out of 5 points of full subscore. In the performance of more complex tasks, visuomotor organization or construction abilities integrate brain functions related to visual and spatial perception with motor execution (33). After a stroke, ADL, such as personal hygiene or dressing, undergo changes in the relationship between arm movements and their visual consequences due to specific lesion damage (34). Additionally, Katz et al. (35), identified a relationship between the visuomotor organization score in LOTCA and the total score of the functional independence measure in 40 patients who had right hemisphere stroke. Furthermore, a previous study highlighted a significant correlation between the visuomotor organization score of the geriatric version of LOTCA and the ADL performance of the MBI in 60 stroke patients within 3 weeks after stroke onset (36). The second decision point of Model II, classifying patients with visuomotor organization scores ≤ 21 points, was the time orientation score of ≤ 1 point in MMSE. Notably, in a previous study involving 95 elderly individuals with minimal cognitive impairment, the orientation score of the clinical dementia rating scale was identified as a strong predictor for progression to Alzheimer’s disease (37). Furthermore, previous studies have indicated that cognitive impairment in orientation following a stroke is linked to a higher risk of recurrent stroke (38), functional ambulation score (39), and disruption of ADL (40). Consistent with these findings, our study suggests that the management of cognitive impairment, especially in visuomotor organization and the time orientation domain, is a key factor in stroke rehabilitation. Furthermore, our results propose cut-off scores that can guide clinicians in considering the specific level of early cognitive function in each stroke individual to set therapeutic goals. Consequently, the outcomes of Model II in our study encourage clinicians to contemplate early cognitive rehabilitation strategies focused on visuomotor organization and orientation, with scores from specific cognitive assessments for predicting good ADL outcomes.

Previous trials showed the possibility to predict independence in ADL after stroke. Research on middle cerebral artery infarct conducted logistic regression analyses using 2 models and reported clinical variables, age, and ADL ability in the second week as good predictors (77% sensitivity) without the added value of neuroimaging variables (41). Also, another clinical study demonstrated that their multivariable models for predicting dependency in ADL during the first year after stroke showed sensitivity of approximately 70% (42). The present study demonstrated 85% sensitivity with overall accuracy of 92% on Model I for predicting ADL on discharge based on motor function at the beginning of hospitalized rehabilitation, which achieved higher performance than previous studies. Meanwhile, Model II for predicting ADL on discharge based on cognitive function at rehabilitation admission exhibited a sensitivity of 63% with overall accuracy of 83%, showing lower predictive capability. However, the specificity revealed 91% and negative predictive value and overall accuracy showed over 80% of performances in Model II , which indicates robustness in accurately identifying the poor outcome group.Nevertheless, the relatively low sensitivity suggests that Model II may have limitations in identifying good outcome candidates.

To the best of our knowledge, this is the first study to investigate both early motor and cognitive functional factors predicting basic ADL outcomes on discharge while proposing the specific cut-off score in each functional assessment. Our contribution to closing the knowledge gap lies in providing empirical values to which clinicians may refer in predicting outcomes and establishing therapeutic rehabilitation goals. This focus on early, predictive functional factors is where our study adds value, offering a clearer direction for enhancing stroke rehabilitation practices based on measurable early interventions. Clinicians might prioritize interventions focusing on sitting-to-standing motor tasks and visuomotor organization functions with specific standardized scoring of functional assessment to enhance basic ADL performance on discharge. This approach not only facilitates targeted rehabilitation strategies but also supports clinicians making informed decisions to optimize patients’ outcome. Furthermore, the results suggest the potential selection of candidates for neuroimaging biomarkers, such as the region of interest in functional near-infrared spectroscopy and tractography in diffusion tensor imaging, to predict basic ADL in stroke patients.

### Limitations

Nevertheless, this study has some limitations. First, the relatively short follow-up period limits our understanding of long-term outcomes, and future research should consider a cohort study with an extended follow-up duration. Second, due to the limitations of the software in handling imbalanced data without the supplementary capabilities of SPSS Modeler, we were unable to directly address data imbalance in our analysis. Future research should consider employing methodologies or tools capable of effectively managing data imbalances to enhance the accuracy and reliability of the results. Third, the specific levels of stroke severity in our sample and relatively small sample size hindered the interpretation of results concerning the generalizability of research findings to a broad stroke population. Although the findings of our study could be applied in the clinical settings for patients with moderate to severe stroke, there would be difficulties in generalizing our findings to stroke survivors with different severity levels. Fourth, we relied solely on the cross-validation technique without external or internal validation and did not utilize a variety of methods such as pruning or generalization to minimize overfitting in the decision tree model. In the future, clinical research should endeavour to employ various overfitting prevention techniques to enhance the validity and reliability of the research findings.

### Conclusion

This study employed the CART algorithm to identify early functional factors predicting the basic ADL outcomes after stroke. Our models underscore the ability to perform sitting-to-standing and visuomotor organization functions at the onset of rehabilitation as the most significant predictors for achieving successful independence in ADL on discharge following a stroke. The inclusion of early motor and cognitive functions in predicting outcomes provides valuable information for clinicians to guide therapeutic strategies.

## Supplementary Material

EARLY FUNCTIONAL FACTORS FOR PREDICTING OUTCOME OF INDEPENDENCE IN DAILY LIVING AFTER STROKE: A DECISION TREE ANALYSIS

EARLY FUNCTIONAL FACTORS FOR PREDICTING OUTCOME OF INDEPENDENCE IN DAILY LIVING AFTER STROKE: A DECISION TREE ANALYSIS

EARLY FUNCTIONAL FACTORS FOR PREDICTING OUTCOME OF INDEPENDENCE IN DAILY LIVING AFTER STROKE: A DECISION TREE ANALYSIS
