# Supplementary material for: EARLY FUNCTIONAL FACTORS FOR PREDICTING OUTCOME OF INDEPENDENCE IN DAILY LIVING AFTER STROKE: A DECISION TREE ANALYSIS
Source: J Rehabil Med. 2024 May 7;56:35095. doi: 10.2340/jrm.v56.35095 (PMC11093115; doi:10.2340/jrm.v56.35095)
Supplement: EARLY FUNCTIONAL FACTORS FOR PREDICTING OUTCOME OF INDEPENDENCE IN DAILY LIVING AFTER STROKE: A DECISION TREE ANALYSIS [file JRM-56-35095-s1.pdf]

Supplementary material has been published as submitted. It has not been copyedited, or typeset by Journal of Rehabilitation Medicine

Table SI. Model performances of the classification tree of Model III

**Model Performance**	**Model III**
**Sensitivity**	0.87
**Specificity**	0.96
**Positive predictive value**	0.89
**Negative predictive value**	0.95
**Overall accuracy**	0.93
