# Supplementary material for: EARLY FUNCTIONAL FACTORS FOR PREDICTING OUTCOME OF INDEPENDENCE IN DAILY LIVING AFTER STROKE: A DECISION TREE ANALYSIS
Source: J Rehabil Med. 2024 May 7;56:35095. doi: 10.2340/jrm.v56.35095 (PMC11093115; doi:10.2340/jrm.v56.35095)
Supplement: EARLY FUNCTIONAL FACTORS FOR PREDICTING OUTCOME OF INDEPENDENCE IN DAILY LIVING AFTER STROKE: A DECISION TREE ANALYSIS [file JRM-56-35095-s2.pdf]

Supplementary material has been published as submitted. It has not been copyedited, or typeset by Journal of Rehabilitation Medicine

Appendix S1

SUPPLEMENTARY MATERIAL

1. Description of clinical assessments

1.1 Range of motion and muscle strength measurements

Passive range of motion (ROM) was measured bilaterally for the following movements of each joint: flexion/abduction of the shoulder, flexion/extension of the elbow and wrist, flexion/extension/abduction/external rotation/internal rotation of the hip, flexion/extension of the knee, and dorsi/plantar flexion of the ankle using a goniometer according to standard techniques [1]. Muscle strength was assessed through a standardized physical examination, and a manual muscle test (MMT) was performed on eight bilateral muscle groups in the upper extremities (shoulder flexor and abductor; elbow and wrist flexors and extensors; gross finger flexors and extensors) and eight bilateral muscle groups in the lower extremities (hip flexor, extensor, abductor, and adductor; knee flexor and extensor; ankle dorsiflexor and plantar flexor). The grading system, ranging from 'zero' to 'normal' according to Medical Research Council (MRC) Scale, was converted to numerical scoring according to the previous study methods to evaluate the recovery from paralysis [2] as follows: 'Zero' to 0, 'Trace' to 5, 'Poor' to 25, 'Poor+' to 35, 'Fair-' to 40, 'Fair' to 50, 'Fair+' to 60, 'Good-' to 70, 'Good' to 80, and 'Normal' to 100. Thus, the summed MMT scores ranged from 0 to 800 for each upper and lower extremity.

1.2 Motor assessment scale

The gross motor functional level of each patient was measured using the motor assessment scale (MAS) by the treating physiotherapist. The MAS is used to evaluate functional recovery of stroke patients including eight items, each scored on a 0 to 6 scale [3]. Higher scores refer to better quality of movement patterns and complexity of the tasks performed. Rolling, lying to sit, sitting balance, sitting-to-standing, walking, upper extremity function, hand movements, and advanced hand activities were included in the MAS. Depending on the patient's functional ability, it takes 10 to 30 min to administer.

1.3 Berg balance scale

Berg Balance Scale (BBS) was used to measure various static and dynamic functional capabilities in sitting and standing. The BBS comprises 14 items, including standing and sitting unsupported, reaching forward, and placing the alternate foot on a stool [4]. Each task was rated from 0 ("unable to perform or requiring help") to 4 ("normal performance"), with a total maximum score of 56 points.

1.4. Rivermead mobility index

The Rivermead mobility index (RMI) is a standardized assessment comprising 15-items that measure functional mobility during gait, balance, and transfers [4]. Performance is scored by a dichotomous (yes/no) scale, yes indicates '1' and no indicates '0'. The maximum score is 15, with higher scores indicating better functional mobility. Among the 15 items, 14 were related to self-reported performance, and one was assessed by the rater through direct observation [4].

1.5. Trunk impairment scale

The trunk impairment scale (TIS) was utilized to assess the ability to control core muscles [5]. This clinical method has demonstrated impairment of core control as well as moving and statistical equilibrium in the sitting position in stroke patients. The TIS has 17 provisions: static sitting balance, 3; dynamic sitting balance, 10; and core's coordinated abilities, 4. Score on the TIS ranges from 0 to 23, with higher points indicating better core control ability.

1.6. Functional ambulatory category

The functional ambulatory category (FAC) is a visual measurement employed to categorize patients based on the fundamental motor skills essential for functional ambulation [6]. The FAC delineates six levels of walking ability. determined by the degree of physical support needed, spanning from 'unable to walk (FAC 0)' to 'able to walk independently anywhere, including stairs (FAC 5)'.

1.7. Fugl-Meyer Assessment

The Fugl-Meyer Assessment (FMA) is a comprehensive quantitative measurement used to assess sensorimotor impairment after a stroke, focusing on voluntary limb movement [7]. Both the motor and sensory components of the FMAs are scored on a 3-point ordinal scale (0–2). The FMA upper extremity (FMA-UE) evaluates reflex activity and voluntary movements within, partially out, and independent synergies. This scale comprises 33 items divided into four subscales: shoulder/elbow (18 items), wrist (five items), hand (seven items), and coordination/speed (three items). Each item is scored on an ordinal 3-point scale, with 2 points awarded for full performance, 1 point for partial performance, and 0 points when the movement cannot be executed. The total score of 66 points indicates full performance on the FMA-UE.

1.8. Mini-Mental State Examination

The Mini-Mental State Examination (MMSE) was employed to assess cognitive function [8]. The scores on the MMSE range from 0 to 30, covering seven cognitive domains: orientation for time and place (each score

range of 0-5), memory registration (0-3), recall (0-3), concentration and calculation (0-5), language function (naming, repetition, three-stage verbal command, and writing) (0-8), and visuospatial construction (0-1).

1.9. Loewenstein occupational therapy cognition assessment

Loewenstein occupational therapy cognition assessment (LOTCA) is a cognitive battery commonly used in neurological rehabilitation settings, comprising 26 items distributed across seven subscales[9] . Each subscale item is rated on a 4 or 5-point scale. The distinctive items and their composite scoring are as follows, 1) Orientation: includes two items - orientation to time and place (scores range from 2 to 16), 2) Visual perception: encompasses four items: object identification, shape identification, overlapping figures, and object constancy (scores rage from 4 to 16), 3) Spatial perception: involves three items - direction on the client's body and spatial relation in pictures (scores range from 3 to 12), 4) Motor praxis: comprises three items - motor imitation, utilization of objects, and symbolic actions (scores range from 3 to 12), 5) Visuomotor organization: examines perceptual-motor integration with spatial components and includes seven items - copying geometric forms, reproduction of two-dimensional models, pegboard construction, colored block designs, reproduction of a puzzle, and drawing a clock (scores range from 7 to 28), 6) Thinking operations: Assesses categorization and sequencing through seven items - categorization, object classification (unstructured and structured), pictorial sequencing, geometric sequencing, and logic questions (scores from 7 to 31), and 7) Attention and concentration: score in this category ranges from 1 to 4.

References

1. Clarkson, H.M., *Musculoskeletal assessment: joint range of motion and manual muscle strength.* 2000: Lippincott Williams & Wilkins.
2. Min, K., et al., *Umbilical cord blood therapy potentiated with erythropoietin for children with cerebral palsy: a double-blind, randomized, placebo-controlled trial.* Stem Cells, 2013. **31**(3): p. 581-591.
3. Brauer, S.G., et al., *Prediction of discharge destination after stroke using the motor assessment scale on admission: a prospective, multisite study.* Archives of physical medicine and rehabilitation, 2008. **89**(6): p. 1061-1065.
4. Berg, K., et al., *Measuring balance in the elderly: preliminary development of an instrument.* Physiotherapy Canada, 1989. **41**(6): p. 304-311.
5. Verheyden, G., et al., *The Trunk Impairment Scale: a new tool to measure motor impairment of the trunk after stroke.* Clinical rehabilitation, 2004. **18**(3): p. 326-334.
6. Holden, M.K., et al., *Clinical gait assessment in the neurologically impaired: reliability and meaningfulness.* Physical therapy, 1984. **64**(1): p. 35-40.

7. Fugel-Meyer, A., et al., *The post-stroke hemiplegic patient1, a method for evaluation of physical perpormance.* Scand. J. Rahabil. Med, 1975. **7**: p. 13-31.
8. Folstein, M.F., L.N. Robins, and J.E. Helzer, *The mini-mental state examination.* Archives of general psychiatry, 1983. **40**(7): p. 812-812.
9. Katz, N., et al., *Loewenstein Occupational Therapy Cognitive Assessment (LOTCA) battery for brain-injured patients: reliability and validity.* The American Journal of occupational therapy, 1989. **43**(3): p. 184-192.
