# Supplementary material for: EARLY FUNCTIONAL FACTORS FOR PREDICTING OUTCOME OF INDEPENDENCE IN DAILY LIVING AFTER STROKE: A DECISION TREE ANALYSIS
Source: J Rehabil Med. 2024 May 7;56:35095. doi: 10.2340/jrm.v56.35095 (PMC11093115; doi:10.2340/jrm.v56.35095)

Supplementary material has been published as submitted. It has not been copyedited, or typeset by Journal of Rehabilitation Medicine

Figure 1. The model III for predicting outcome of ADLs at discharge by early cognitive functions. MAS, motor assessment scale; BBS, berg balance scale; MMT, manual muscle test; LOTCA, Lowenstein occupational therapy cognitive assessment

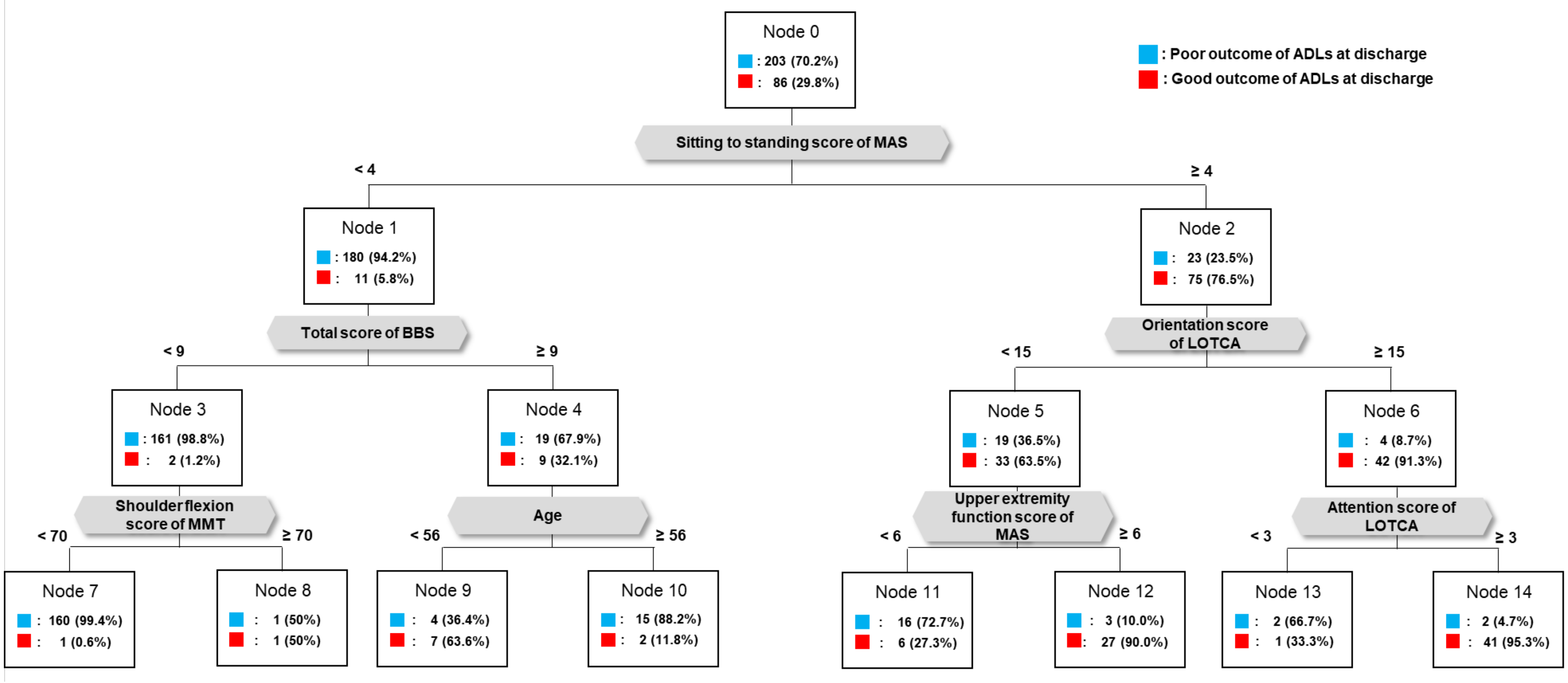

Supplement: EARLY FUNCTIONAL FACTORS FOR PREDICTING OUTCOME OF INDEPENDENCE IN DAILY LIVING AFTER STROKE: A DECISION TREE ANALYSIS [file JRM-56-35095-s3.pdf]
